# Falls in People with Multiple Sclerosis Compared with Falls in Healthy Controls

**DOI:** 10.1371/journal.pone.0107620

**Published:** 2014-09-25

**Authors:** Rajarshi Mazumder, Charles Murchison, Dennis Bourdette, Michelle Cameron

**Affiliations:** 1 Oregon Health & Science University, Portland, Oregon, United States of America; 2 Department of Neurology, Oregon Health & Science University, Portland, Oregon, United States of America; 3 Portland Veteran Administration Medical Center, Portland, Oregon, United States of America; University of Oxford, United Kingdom

## Abstract

**Objective:**

To compare the risk, circumstances, consequences and causes of prospectively recorded falls between people with multiple sclerosis (PwMS) and healthy controls of similar age and gender.

**Methods:**

58 PwMS and 58 healthy controls, who are community-dwelling, were recruited in this 6-month prospective cohort study. 90% of PwMS and 84% of healthy controls completed the study. Participants counted falls prospectively using fall calendars and noted fall location, fall-related injuries, and the cause of the falls. Kaplan Meier survival analysis and log-rank tests were performed to compare the distributions of survival without falling between PwMS and healthy controls.

**Results:**

40.8% of controls and 71.2% of PwMS fell at least once. 48.1% of PwMS and 18.4% of healthy controls fell at least twice. 42.3% of PwMS and 20.4% of health controls sustained a fall-related injury. After adjusting for age and gender, the time to first fall (HR: 1.87, p = 0.033) and the time to recurrent falls (HR: 2.87, p = 0.0082) were significantly different between PwMS and healthy controls. PwMS reported an almost equal number of falls inside and outside, 86% of the falls in healthy controls were outside. Healthy controls were more likely to fall due to slipping on a slippery surface (39.5% vs 10.4%). PwMS more often attributed falls to distraction (31% vs 7%) and uniquely attributed falls to fatigue or heat.

**Conclusions:**

Fall risk, circumstances, consequences, and causes are different for PwMS than for healthy people of the same age and gender. PwMS fall more, are more likely to be injured by a fall, and often fall indoors. PwMS, but not healthy controls, frequently fall because they are distracted, fatigued or hot.

## Introduction

People with multiple sclerosis (PwMS) have a high incidence of falls. [1]–[4] Studies demonstrate that over 50% of people with Multiple Sclerosis (MS) fall in a three to six-month period and around 30 to 50% fall multiple times. [1], [5], [6] Falls in people with MS are associated with injury [3], [7] and death. [8] Falls also negatively impact quality of life in individuals with MS causing fear of falling, reduced activities, and reduced community participation [9].

Healthy adults also fall. Particularly, older adults fall frequently and suffer significant consequences from these falls. As a result, falls in older adults have been studied extensively. [10] However, little is known about how the frequency, circumstances and consequences of falls in adults of a similar age and gender distribution to PwMS, who are young and predominantly female. In addition, while studies indicate either that men with MS are at higher risk for falls than women with MS, [2], [3] or that there is no effect of gender on fall risk in MS, [1], [11] one study in Veterans with MS found that injurious falls were significantly more common in women with MS than in women without MS but that there was not a significant difference in the risk for injurious falls in men with MS compared to men without MS [12].

The purpose of this study was to compare the incidence, circumstances, consequences and causes of prospectively recorded falls in a cohort of people with MS with the incidence, circumstances, consequences and causes of prospectively recorded falls in a cohort healthy control subjects.

## Methods

**Figure 1:**
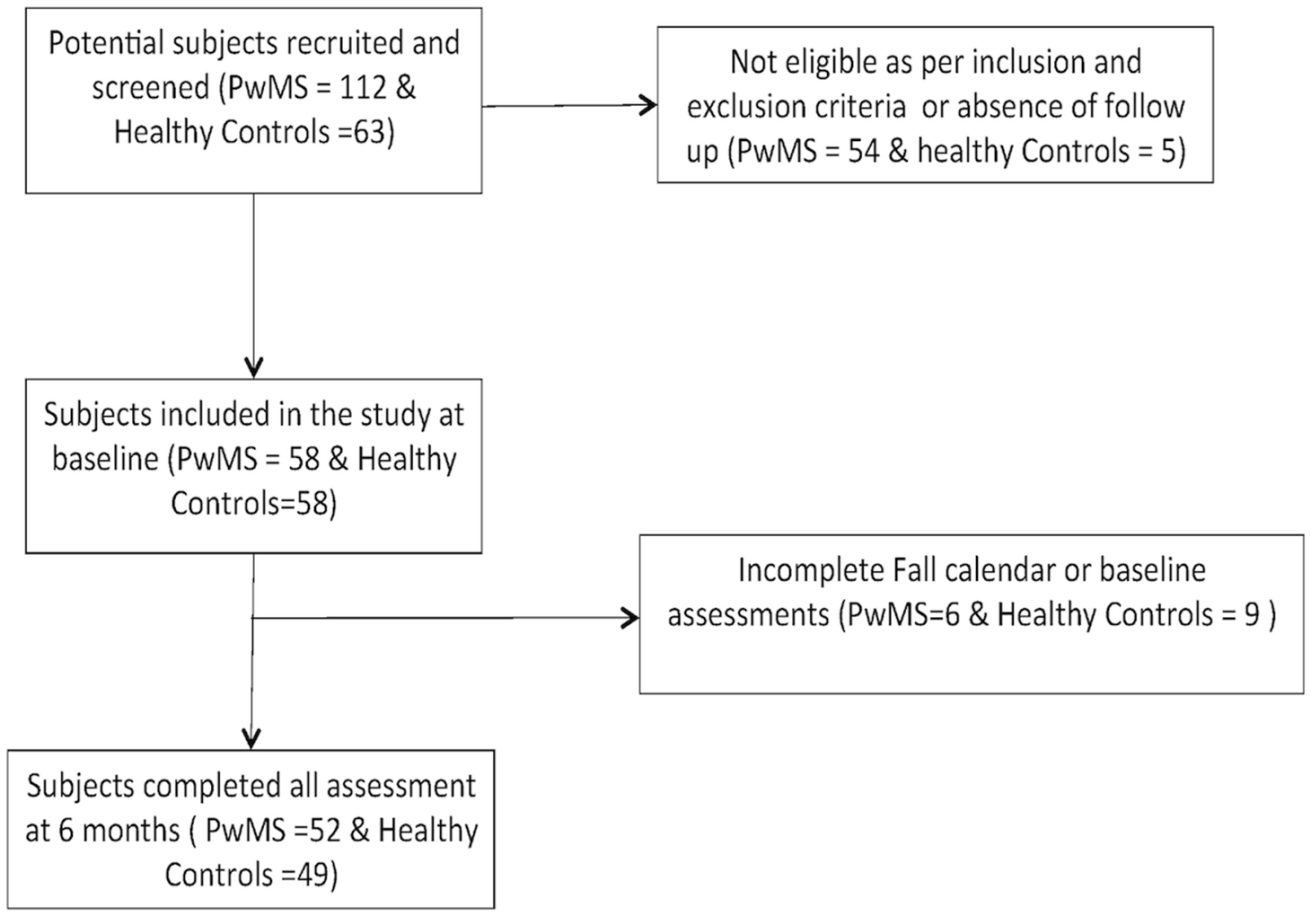
Participant flow diagram.

This was a prospective cohort study carried out at a Department of Veterans Affairs and an academic medical center in the Northwest USA. The Institutional Review Board of the Portland VA Medical Center and Oregon Health & Science University approved this study and the investigation was conducted according to the principles expressed in the Declaration of Helsinki. Written informed consent was obtained from the participants. The subjects with MS were recruited from the outpatient MS specialty clinics of these centers and surrounding community neurology clinics in 2010 to 2011. The clinics serve a total of approximately 1400 people with MS annually. Potential subjects were recruited using flyers posted at the clinics, by providing information at patient education programs and support groups, and by referral from clinic healthcare providers. The healthy controls were people who responded to posted fliers. 112 potential subjects with MS and 63 potential healthy controls expressed interest in participating in the study and, of these, 58 with MS and 58 healthy controls met inclusion criteria and consented to participate. 52 subjects with MS and 49 healthy controls completed all measures analyzed and were included in this analysis (Figure 1).

### Inclusion and Exclusion Criteria

Inclusion criteria for both PwMS and the healthy controls were age from 18 to 50 years, willing and intellectually able to understand and sign an informed consent and adhere to protocol requirements, able to complete a written daily record of falls for 6 months and community dwelling. The PwMS also had to have a clinically and MRI-confirmed diagnosis of MS [13] of any subtype, mild-to-moderate MS-associated disability [14] and no clinically significant MS relapse within 30 days prior to baseline testing. Exclusion criteria were a self-reported condition other than MS known to affect balance or gait, unable to follow directions in English, unhealed fractures or other conditions conveying risk of injury during balance testing; blindness, or, unable to walk more than 100 meters.

### Variables

#### Falls

Falls and injurious falls in the 6 months following the baseline assessment were assessed prospectively by subjects documenting their falls each day for 6 months on monthly fall calendars and returning these calendars at the end of each month. The calendar stated, “Please write in the number of falls you have each day. A fall is any unexpected event that results in you ending up on the ground, floor, or any lower surface.” [15], [16] Participants also documented each month if they suffered any injuries, including bruise/cut/graze/pain, sprain/strain, or other injuries, as a result of a fall. In addition, for the first two falls each month, the participants recorded details about the location of the fall, activities performed at the time of the fall and any injuries sustained as a consequence of the fall. Subjects were contacted by phone during the first week of the fall count to reminder them to count their falls and to answer any calendar-related questions. Subjects returned the completed fall calendar at the end of each month and if the calendar was not received within 1 week after the end of the month, subjects were contacted by phone to ask for the calendar to be sent in.

### Statistical Methods

Demographic characteristics of the participants were summarized using mean, standard deviations, range or frequencies. Fall counts and fall rates (Falls per person/month = (Total no. of falls/Total no. of person-days)×180 days), number of fallers (at least 1 fall in the 6 months), number of recurrent fallers (two or more falls in the 6 months) and injurious falls were compared between PwMS and healthy controls. Difference between demographic characteristics and proportion of fallers, recurrent fallers and injurious were assessed using Mann-Whitney U test and Fisher exact test as indicated.

Time to first fall and time to recurrent falls (second fall) was operationalized as the number of days enrolled in the study to the event. Kaplan Meier survival analysis and log-rank tests were performed for time to first fall and time to recurrent fall to compare the distributions of survival without falling between PwMS and healthy controls. Cox proportional hazards model was then used to test whether time to first fall and time to recurrent fall were significantly different between PwMS and healthy controls after adjusting for age and gender.

The frequency of the total number of recorded falls in each category of the circumstances (i.e. inside, outside, inside (while standing, turning, walking) and self-perceived causes (i.e. lost balance, trip over something) were determined. In addition, frequency and type of injurious falls were determined. Injurious falls were classified as bruise/cut/graze/pain, sprain/strain, or others. Hypothesis testing was not performed to assess the difference between the circumstances, self-perceived causes and injurious consequences of falls in people with MS and healthy controls due to low cell counts and lack of power.

STATA 12.1 (StataCorp, Texas) and R (version 2.15.3) were used for statistical analysis and all hypotheses were tested using 2-sided tests with the significance level set at 5%.

## Results

### Participant Characteristics

All analyses are based on data from the 52 participants with MS and 49 healthy controls who returned all fall calendars and had complete demographic information. There were no statistically significant differences in demographic characteristics between the 101 participants with complete data and the 15 participants with insufficient data for inclusion.

Demographics and MS disease characteristics of the study participants are summarized in Table 1. The average age of PwMS was 39.7 years, with a range of 22 to 50 years, and the average age of the people in the control group was 38.7 years, with a range of 18 to 50 years. 67% of the participants with MS and 80% of the healthy controls were female and 94% of the participants with MS had RRMS. This age and gender distribution is typical for people with MS. The participants’ average duration of disease was 6.1 years and their median Expanded Disability Status Scale (EDSS) was 3.0.

**Table 1:** Demographics of PwMS and healthy control subjects.

	PwMS (n = 52)	Controls (n = 49)	p-value
Age (mean, s.d, range)	39.7, 8.4, 22–50	38.7, 9.5,18–50	0.783
Sex (% female)	67%	80%	0.184
EDSS (median, range)	3.0, 0–6	N/A	
Disease duration (years,mean, s.d., range)	6.1, 5.4, 1–22	N/A	

### Falls Incidence

**Figure 2:**
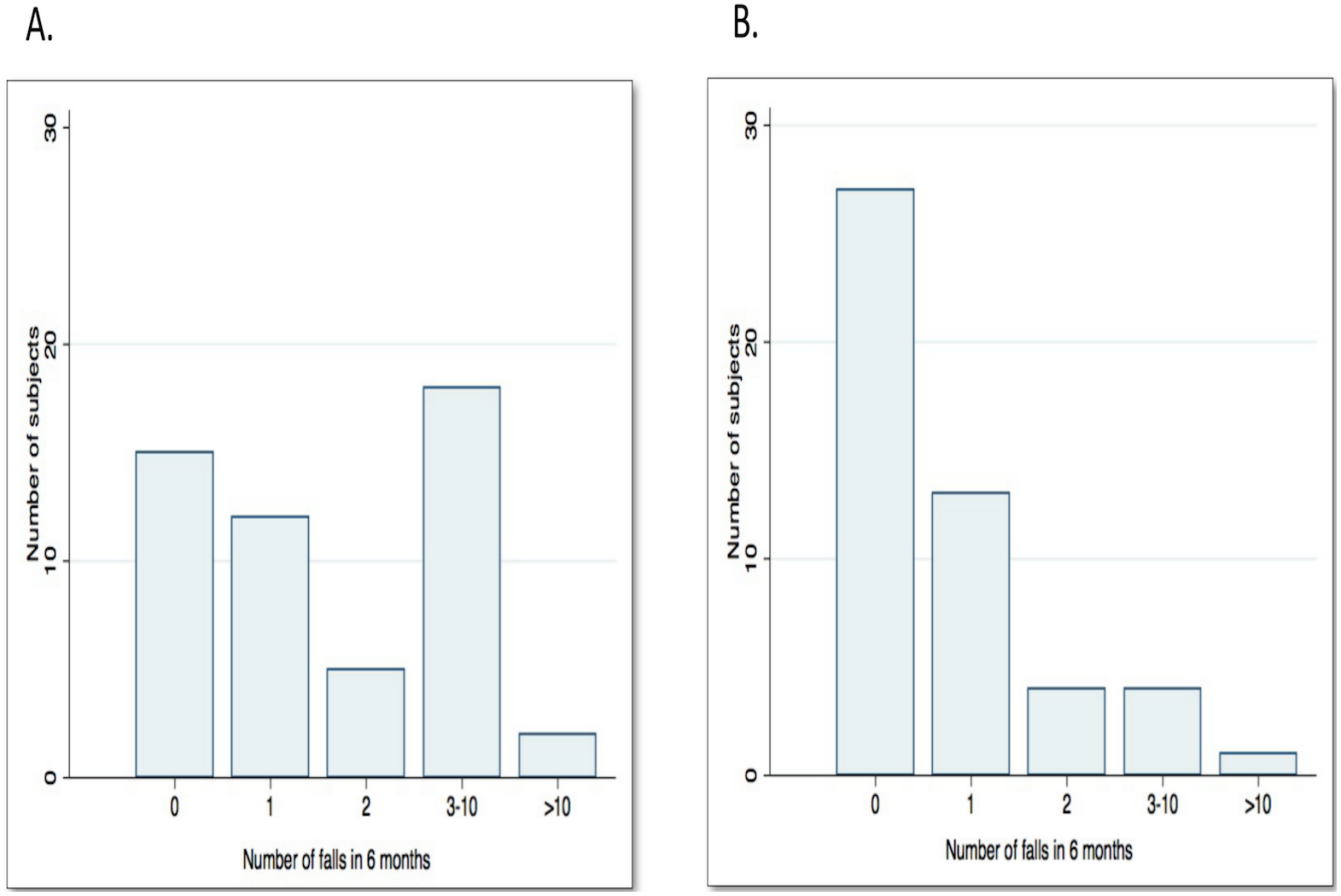
Frequency of falls in participants with MS and healthy controls. (A) in participants with MS (n = 52, No falls in 28.8%, 1 fall in 23.1%, 2 falls in 9.6% and 3–10 falls in 34.6%); (B) in healthy controls (n = 49, No falls in 57.1%, 1 fall in 24.5%, 2 falls in 8.2%, 3–10 falls in 8.2%).

A total of 200 falls were recorded, 145 in the participants with MS and 55 in the healthy controls. PwMS fell more than the healthy controls. They had significantly more falls in the entire 6 month period, they had a higher fall rate (falls/person/month), more were fallers and recurrent fallers, and more sustained injuries from falls (Table 2, Figure 2).

**Table 2:** Number of falls in PwMS and healthy controls.

	PwMS (n = 52)	Controls (n = 49)	p-value, comparison
No. of falls in 6 months(mean, SD, range)	2.79, 3.96, 0–21	1.20, 2.49, 0–15	0.0007
Fall rate (falls/month/person)(mean, SD, Range)	0.46, 0.65, 0–3.5	0.19, 0.41, 0–2.5	0.0007
Proportion of fallers(1 or more falls in the6 month period)	37 fallers/52 = 71.2%	20 fallers/49 = 40.8%	0.004
Proportion of recurrent fallers(2 or more falls inthe 6 month period)	25 recurrent fallers/52 = 48.1%	9 recurrent fallers/49 = 18.4%	0.002
Proportion of injuriousfallers (1 or more injuriousfalls in the 6 month period)	22 injurious fallers/52 = 42.3%	10 injurious fallers/49 = 20.4%	0.018

### Time to First Fall and Time to Recurrent Falls

**Figure 3:**
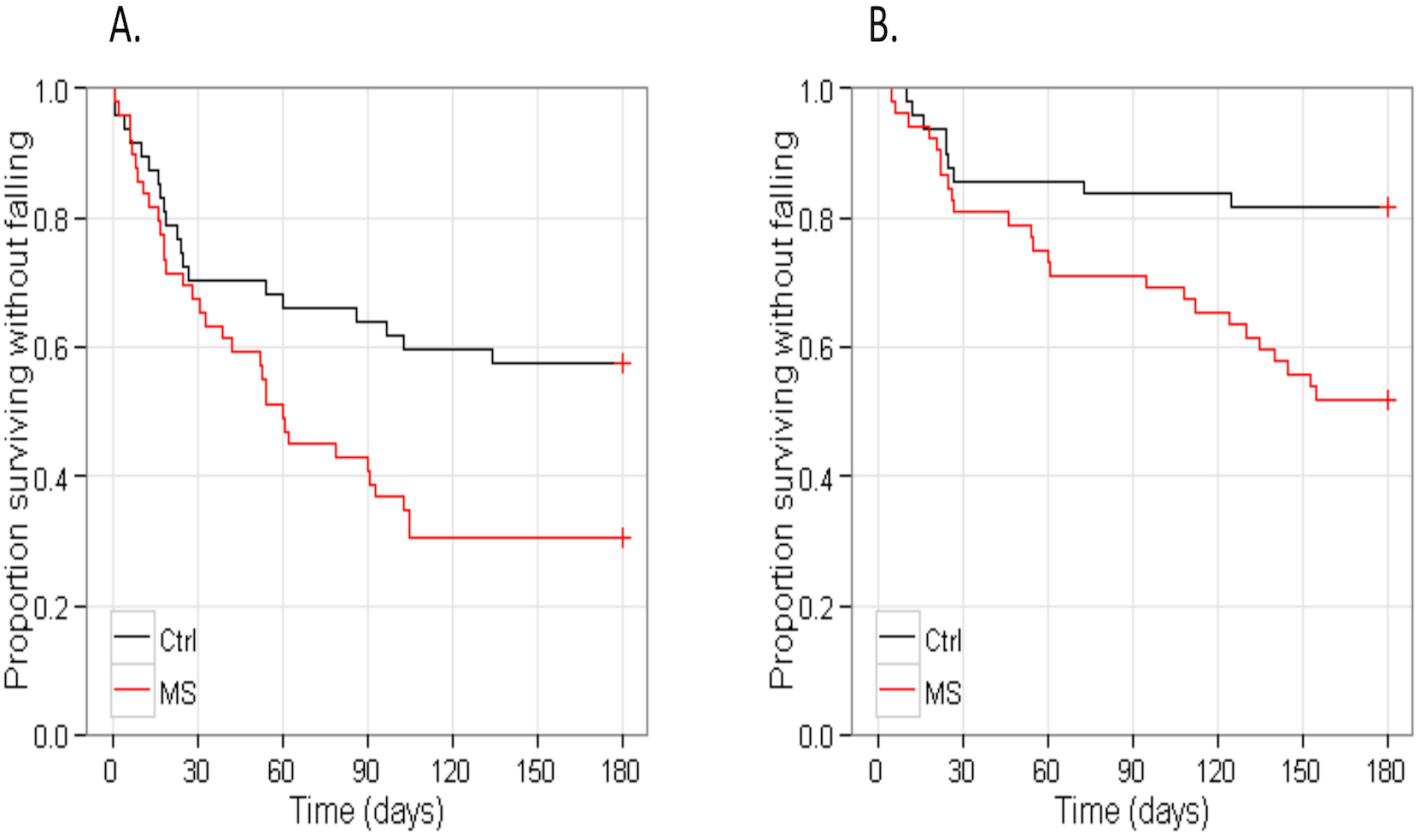
Kaplan-Meier survival curves for A) Time to first fall, and B) Time to recurrent falls in people with MS and healthy controls.

Univariate Cox analysis showed that the time to first fall (Hazard Ration (HR): 1.95, p = 0.018) and the time to recurrent falls (HR: 2.99, p = 0.0049) were significantly different between PwMS and the healthy controls. (Figure 3) After adjusting for age and gender, the time to first fall (HR: 1.87, p = 0.033) and the time to recurrent falls (HR: 2.87, p = 0.0082) remained significantly different between PwMS and the healthy controls.

### Circumstances and Consequences of Falls

Of the 200 falls recorded, information about the circumstances (location and activity) and consequences (associated injuries) of the falls were recorded for 168 falls, 125 in participants with MS and 43 in the healthy controls. These were the first 2 falls per person each month.

The locations and activities associated with falls were different for PwMS than for healthy controls. PwMS reported an almost equal number of falls inside and outside. In contrast, among healthy controls, 86% of the falls were outside. Most of the falls among PwMS occurred while standing, turning or walking inside (26.4%) or while performing basic tasks of everyday life such as getting on or off chair (12%), climbing up or down stairs (8.8%), or outside the house on the lawn (7.1%). In contrast, the healthy controls mostly fell away from the house (76.7%) or inside climbing stairs (9.3%) (Table 3).

**Table 3:** Fall locations for people with MS and healthy controls.

	MS (n = 52, falls = 125) number of falls,(% of total recorded falls)	Ctrl (n = 49, falls = 43), number of falls,(% of total recordedfalls)
**Falls inside**	60 (48%)	6 (14%)
**Falls outside**	65 (52%)	37 (86%)
**Inside**		
Inside (While standing,turning, walking)	33 (26.4%)	2 (4.6%)
Inside (While gettingon/off chair)	15 (12%)	0 (0%)
Inside (When standing on chair)	1 (0.8%)	0 (0%)
Inside (When climbing up or down stairs)	11 (8.8%)	4(9.3%)
**Outside**		
House entrance/garden (When climbing up/down step)	4 (3.2%)	0
House entrance/garden (Ona path)	4 (3.2%)	3 (6.9%)
House entrance/garden (Onthe lawn)	12 (7.1%)	1 (2.3%)
Away (Walkingup/down stairs)	3 (2.4%)	4 (9.3%)
Away (In the street)	6 (4.8%)	6 (13.9%)
Away (At the shops)	0	0
Away (In a publicbuilding)	4 (3.2%)	3 (6.9%)
Away (At anotherPerson’s house)	3 (2.4%)	3 (6.9%)
Away (Getting in/outof vehicle)	0	0
Away (Other)	31 (24.8%)	17 (39.5%)

Two individuals with MS reported more than ten falls during the six-month period, one of these sustained all of these falls inside the home, generally tripping or slipping. The other mostly fell away from the home, at the office, on the sidewalk or at the park. In contrast, the only healthy control subject who reported more than 10 falls sustained all these falls on one day while jumping on a trampoline.

A higher proportion of the PwMS (42.3%) than of the healthy controls (20.4%) sustained at least one fall-related injury. Of the 168 falls where information about consequences was recorded, 50 were associated with injury. Thirty eight of the 125 (30%) falls in the PwMS and 12 of the 43 (28%) falls in the healthy controls were associated with injury. These injuries were classified as bruise/cut/graze/pain, sprain/strain, or others. In PwMS, 34 of the falls caused bruises, cuts, grazes or pain, 2 of the falls caused sprains or strains and the other consequences included one fall in a person with MS resulting in a collapsed lung and ruptured spleen. Among the healthy controls 9 of the falls caused bruises, cuts, grazes or pain, 3 of the falls caused sprains or strains and none resulted in severe injuries.

### Causes of Falls

Loss of balance, the most common reported cause of falls, was reported by similar proportions of the PwMS (43%) and the healthy controls (37%). Other causes of falls differed for PwMS and healthy controls. Only PwMS reported fatigue (14%) and heat (2%) as causes of falls. PwMS were more likely than healthy controls to report distraction (31% vs 7%) and tripping over something (25% vs 12%) as the cause of falls. Slipping on a slippery surface was more commonly reported among healthy controls (39.5% vs 10.4%).

## Discussion

This is the first study to compare the risk, circumstances, consequences, and causes of falls recorded prospectively among PwMS and healthy controls of similar age and gender. We found that PwMS had a higher risk for falls compared to healthy controls of similar age and gender. PwMS were significantly more likely than healthy controls to be first in experiencing falls and recurrent falls. PwMS fell almost equally inside and outside the house, as compared to healthy controls who fell mostly outside the house. PwMS sustained more fall related injuries. PwMS uniquely identified fatigue and heat as causes of falls and, more often than healthy controls, identified distraction as a cause of falls.

A number of previous studies have prospectively evaluated falls in PwMS. These have found that approximately fifty to seventy percent of their cohorts with MS fell at least once in a three to twelve month period. [3], [4], [6], [17] Our study confirms this high risk for falling in PwMS finding that 70% fell at least once and 48% fell at least twice in 6 months. This was despite our cohort being younger and less disabled than in most previous studies. None of these prior studies compared these fall rates with fall risks in healthy people of similar age and gender. We found that although falls do occur in healthy adults under the age of 50, the number of falls, the frequency of falls, the risk for falls, the proportion of recurrent fallers and the proportion of injurious fallers in PwMS were all significantly higher. In addition, we found that PwMS were approximately 67% and 75% more likely to be first in experiencing both single fall and recurrent falls, respectively, compared to healthy controls of similar age and gender.

Only one previous study has reported on the location, circumstances and consequences of falls in PwMS captured prospectively. [3] They found that most falls occurred inside the house while performing task related to activities of daily living and general mobility (standing, turning, and walking). In our study we found that PwMS fell approximately equally inside and outside the house and were also associated with activities of daily living and general mobility. In our population without MS most falls (86%) occurred outside. It is likely that our MS cohort had more falls outside than the previously studied cohort because our cohort was younger and less disabled, and therefore may be spending more time outside. Our study found that approximately 30% of the falls in PwMS and healthy controls were associated with injury, most commonly bruises, cuts and sprains. The similar risk for injury in people with and without MS in our study but the higher overall incidence of fall-related injuries in the PwMS suggests that the higher risk for injury in PwMS is because PwMS fall so often. In comparison, Gunn et al found only 11% of the falls in their cohort with MS were associated with reports of injuries. [3] This lower risk of injury may be because their older and more disabled cohort took less risk and because most of the falls occurred at home which may be safer than outside.

The causes for falls in PwMS found in this study are similar to those reported in previous studies. Similar to prior studies, [11], [18], [19] loss of balance was the most common cause of falls in PwMS. This was also the most common cause of falls in the healthy controls. Our study found that in PwMS 35% of falls occurred due to trips or slips. This is similar to results found in a cross-sectional study by Matsuda et al, [5] where 48% of falls in PwMS occurred due to trips or slips while walking. Fatigue and heat, which were the unique causes of falls in our cohort with MS, are also similar to a prior study which found that PwMS attribute their falls to MS-related symptoms such as fatigue, heat sensitivity and loss of vision. [20] In addition, we confirmed that distraction is a common cause for falls in PwMS. This was found to rarely be a cause for falls in the healthy controls.

Overall, our study demonstrates that falls in younger adults with MS are different from falls in people without MS of similar age and gender. Young adult PwMS with a low level of disability fall more than people without MS of a similar age, they are more likely to fall inside the home, and they have different causes for falls, including fatigue, heat, and distraction. They have a similar risk for injury with each fall, although they are more likely to be injured by a fall because they fall so often. These fall characteristics are also different from reported characteristics of falls in older adults. The risk for falls reported in PwMS of any age is higher than that reported for older adults. Approximately one third of older adults fall in one year compared to approximately 70% of PwMS falling in 6 months in our study. [10], [21] Similar to older adults, soft tissue injuries such as cuts, grazes or bruises [22] were the most common injuries in PwMS. Studies with larger cohorts are needed to assess the risk for serious injury and mortality. Even in older adults, only 5% of falls induce fracture or require hospitalization [22].

This study has a number of strengths. To date it is the first study to compare the risk of falls in PwMS with healthy controls of similar age and gender. Understanding the differential risk of fall in PwMS as compared to individuals of similar age and gender could be useful in suggesting unique interventions to prevent fall-related adverse outcomes in PwMS, who experience a significantly high frequency of falls. Prospective assessment of falls in both PwMS and healthy controls allows comparison of time to first fall using Cox proportional models, which thus includes not just whether someone fell but also assess the odds of one group experiencing a fall faster than the other. Fall frequency, circumstances and causes were collected prospectively using a fall calendar, which is the current gold standard to assessing falls. This limits overestimation of falls and decreases bias from misclassification.

### Study Limitations

This study was powered by the expected number of falls in PwMS. The small number of falls in the healthy controls limited some comparative hypothesis driven statistical analyses and interpretation. In the future, comparative studies should be designed to account for fewer falls in the healthy controls. This study focused on younger people with MS whose falls may differ from those in older people with MS. Older people may have different past experiences, risk taking behaviors and levels of activity.

## Conclusions

Fall risk, circumstances, consequences, and causes are different for PwMS than for healthy people of the same age and gender. PwMS fall more, are more likely to be injured by a fall, and often fall indoors. PwMS, but not healthy controls, frequently fall because they are distracted, fatigued or hot. Although the risk for injury with each fall is similar for people with or without MS, the risk of fall-related injury is twice as high in people with MS than in people without MS. PwMS, even individuals who are younger and with less disability are at risk increased risk for accidental falls due to unique risk factors and circumstances. Further research is needed to identify the optimal interventions to prevent falls in this population.

## Supporting Information

File S1 Data that were used in this analysis.
(XLSM)
